# Bovine papillomavirus on the scene of crime: is E5 oncogene the only guilty party?

**DOI:** 10.1186/1750-9378-8-26

**Published:** 2013-07-05

**Authors:** Giuseppe Borzacchiello

**Affiliations:** 1 Department of Veterinary medicine and Animal productions, University of Naples Federico II, Via F. Delpino, 1-80137, Naples, Italy

**Keywords:** Bovine papillomavirus, E5, E6, E7

## Abstract

Bovine papillomaviruses (BPVs) induce hyperplastic and tumoral lesions not only in cows but also in other different animal species. The transforming activity of BPVs is due to its major E5 oncogene. Recent studies have highlighted the role of E5 in cancer development but very little is known about E6 and E7 oncogenes. In this letter we argue for the need of investigating E6 as well as E7 to better understand the role of these two oncogenes during carcinogenesis.

## Letter to the editor

Bovine papillomavirus (BPV) is considered as causative agent of cutaneous and mucosal tumors in its natural host [1]. However, BPV is the only papillomavirus (PV) able to cross infect other species being associated to tumors also in equids [2] buffaloes [3,4], yaks [5], giraffes [6], tapirs [7], zebras and bison [6,8]. BPV has been also largely investigated as animal model to better understand the transforming activity of Pvs. BPV attracted the interest of molecular biologists since it was the first Pv able to induce transformation in cultured non-epithelial cells; furthermore its genome was the first among PVs to be completely sequenced [9]. Further genetic analysis identified two BPV early (E) genes, E5 and E6, with a direct role in cell transformation [10,11]. E5 was identified as the major BPV oncogene whose transformation activity lies in its binding to and activation of the β subunit of the platelet derived growth factor receptor (PDGFβ-r) [12]. Most if not all these observations were derived from in vitro studies.

In the last decade, the role of BPV major oncoprotein E5 in cell transformation has been largely investigated in naturally occurring bovine and equine tumors. Overall, these studies confirmed the pivotal role of E5 in cancer development supporting the role of the virus [13-18]. Interestingly, new facets of BPV infection and pathogenesis have come to light from recent in vivo studies paving the way for new fields of speculation about PV biology, thus confirming the importance of BPV as animal model [19].

Pv’s contribution to tumorigenesis is “paradigmatically” based on the actions of more than one oncogene. Human Pvs (HPVs) E5, E6 and E7 oncoproteins act synergistically disturbing different cellular pathways and in so doing they contribute to initiation and progression of cancer [20]. Moreover, only the activity of both HPV E6 and E7 immortalize primary culture cells [21].

In this regard, among BPV’s oncogenes the contribution of the other two oncogenes (E6 and E7) to cancer development is less known. It has been suggested that BPV E7 may cooperate with E5 in inducing cell transformation whereas E6 can downregulate p53 transcriptional activity by interacting with CBP/300 [22-24]. However, the contribution of BPV to cancer development, according to “PV’s paradigm”, may not result only from E5 oncoprotein’s activity [25]. More and more studies should investigate BPV E6 and E7 to understand their possible role in animal cancer development in order to better define the molecular scenario of bovine and equids tumors. Moreover, from a comparative point of view, further investigations on BPVs will still give a contribute to discovering new aspects of PV’s biology and pathology.

## Competing interests

The author declares that he has no competing interests.
